# *Eucommia ulmoides* Olive Male Flower Extracts Ameliorate Alzheimer’s Disease-Like Pathology in Zebrafish *via* Regulating Autophagy, Acetylcholinesterase, and the Dopamine Transporter

**DOI:** 10.3389/fnmol.2022.901953

**Published:** 2022-06-09

**Authors:** Chen Sun, Shanshan Zhang, Shuaikang Ba, Jiao Dang, Qingyu Ren, Yongqiang Zhu, Kechun Liu, Meng Jin

**Affiliations:** ^1^Biology Institute, Qilu University of Technology (Shandong Academy of Sciences), Jinan, China; ^2^Key Laboratory for Drug Screening Technology, Shandong Academy of Sciences, Jinan, China

**Keywords:** AD, AlCl_3_, Ache, *slc6a3*, flavonoids

## Abstract

Alzheimer’s disease (AD) is the most prevalent neural disorder. However, the therapeutic agents for AD are limited. *Eucommia ulmoides* Olive (EUO) is widely used as a traditional Chinese herb to treat various neurodegenerative disorders. Therefore, we investigated whether the extracts of EUO male flower (EUMF) have therapeutic effects against AD. We focused on the flavonoids of EUMF and identified the composition using a targeted HPLC-MS analysis. As a result, 125 flavonoids and flavanols, 32 flavanones, 22 isoflavonoids, 11 chalcones and dihydrochalcones, and 17 anthocyanins were identified. Then, the anti-AD effects of the EUMF were tested by using zebrafish AD model. The behavioral changes were detected by automated video-tracking system. Aβ deposition was assayed by thioflavin S staining. Ache activity and cell apoptosis in zebrafish were tested by, Acetylcholine Assay Kit and TUNEL assay, respectively. The results showed that EUMF significantly rescued the dyskinesia of zebrafish and inhibited Aβ deposition, Ache activity, and occurrence of cell apoptosis in the head of zebrafish induced by AlCl_3_. We also investigated the mechanism underlying anti-AD effects of EUMF by RT-qPCR and found that EUMF ameliorated AD-like symptoms possibly through inhibiting excessive autophagy and the abnormal expressions of *ache* and *slc6a3* genes. In summary, our findings suggested EUMF can be a therapeutic candidate for AD treatment.

## Introduction

Alzheimer’s disease (AD) is a common neurodegenerative disease which is age-related. Patients with AD are characterized by the progressive loss of acquired knowledge and memory decline. The loss of neurons, formation of neurofibrillary tangles, tau protein aggregation, amyloid β-protein (Aβ) deposition, and low levels of acetylcholine (ACh) are the main clinical hallmarks of AD (Kepp, 2016; Sanabria-Castro et al., 2017).

The aging tendency of the population is leading to an increased prevalence of AD. Currently, over 47 million people have been diagnosed with AD, and this has caused heavy burdens for families and society (Neves et al., 2021). To deal with this situation, many AD drugs have been developed, such as anti-tau, an amyloid β-protein (Aβ) aggregation inhibitor, and cholinergic-enhancing and anti-inflammatory drugs. Unfortunately, these drugs are not able to prevent the progression of AD and only can improve cognitive function and memory to a certain extent (Pearson, 2001; Huang and Mucke, 2012). Therefore, the development of AD drugs is an urgent task.

Because of their novel structures and extensive physiological activities, natural products from plants have been always an important source of drug development. *Eucommia ulmoides* Olive (EUO), also named Du-zhong, is a deciduous tree in the family of *Eucommiaceae* (Yan et al., 2018). It is also the traditional Chinese herb. The leaf and bark of EUO are officially documented in the Chinese Pharmacopeia. The leaf extracts of EUO are reported to be treat AD, aging, diabetes, hypertension, and osteoporosis (He et al., 2014). However, studies investigating the male flowers of EUO began relatively late. Currently, many studies have shown that the male flowers, like the leaf and bark of EUO, also contain many bioactive components including lignans, megastigmane glycosides, iridoids, phenolics, and flavonoids. These bioactive constituents have typically exhibited neuroprotective, anti-oxidant, anti-tumor, anti-inflammatory, anti-hypertensive, anti-aging, immunity promotion, and other activities (Luo et al., 2010; Kobayashi et al., 2012; Zhang et al., 2012; Niu et al., 2015; Hao et al., 2016; Yan et al., 2018). There are similar bioactive constituents between the male flower and leaf of EUO. Hence, we hypothesize that extracts of the EUO male flower (hereafter referred to as EUMF) may have anti-AD activity.

Zebrafish is an ideal model system for human disease and drug development. They possess a high homology to humans and have rapid development and small sizes (Kerstin et al., 2013; Hong et al., 2020). Many studies have reported that a zebrafish AD model can be established by using AlCl_3_, an *in vivo* animal model that can mirror the primary characteristic pathological changes of patients with AD. Various clinical hallmarks of AD can be detected in this model (Huang et al., 2016; Pan et al., 2019). But unfortunately, an important clinical hallmark-Aβ deposition has not yet been successfully detected in zebrafish AD model.

In summary, in this study we isolated and purified the EUMF and identified the chemical compositions. To verify our hypothesis mentioned in the previous paragraph, the zebrafish AD model was used to investigate the therapeutic effect of EUMF on AD symptoms. In addition, Aβ deposition detection was used innovatively in our zebrafish AD model. Finally, we further tested the mRNA expressions of key factors involved in autophagy and the regulation of neurotransmitters to reveal the underlying mechanism.

## Materials and Methods

### Animals

The adult wild-type zebrafish (AB strain) were maintained in a zebrafish facility at 28.5°C ± 0.5°C with a 14 h light/10 h dark cycle photoperiod at the Key Laboratory for Drug Screening Technology of the Shandong Academy of Sciences. Larvae were obtained from natural mating. Zebrafish larvae at 3 days post-fertilization (dpf) were used this study. All experiments were conducted in compliance with the standard ethical guidelines and under the control of the Biology Institute, the Qilu University of Technology of Animal Ethics Committee.

### Preparation of the *Eucommia ulmoides* Olive Male Flower

The hydrothermal extraction method is used to prepare EUMF. Approximately 20 g of dried EUMF powder was placed into a flask, and 2,000 mL of ultrapure water was added. Then the flask was placed into an electric jacket for extraction by heat reflux three times, 2 h each time. The supernatant was obtained by centrifugation at 5,000 rpm for 10 min. The combined extraction solution was concentrated by rotary evaporation and then freeze-dried to obtain the EUMF.

### Identification of Flavonoid Compounds Using HPLC-MS

A targeted HPLC-MS analysis of the flavonoid compounds was performed on SCIEX Qtrap 6500 + system (SCIEX, United States). The Xselect HSS T3 C_18_ column (2.1 × 150 mm, 2.5 μm) was used for sample separation. Distilled water containing 0.1% formic acid was used as solvent A, and acetonitrile containing 0.1% formic acid was used as solvent B. The elution condition was maintained at 2% B for 2 min, from 2 to 100% B for 13 min, maintained at 100% B for 2 min, and equilibrated with the initial elution solvent for 3 min. The flow rate was 0.4 mL/min. The injection volume of the sample was 1 μL. The column temperature was set to be 50°C. Mass spectrometry was performed in both the positive and negative ion modes. The optimal positive MS parameters were a curtain gas pressure of 35 psig and an ion spray voltage of 5,500 V at a temperature of 550°C. For the negative MS mode, the ion spray voltage was set as −4,500 V and the other parameter was the same as the positive mode. All of the compounds were identified according to LC and MS information and compared with flavonoid compound databases that were supplied by the Novogene Co., Ltd. (Tianjin, China).

### Establishment of Zebrafish Alzheimer’s Disease Model

The establishment of the zebrafish AD model referenced to the previous studies (Huang et al., 2016; Pan et al., 2019) with a slight modification. In brief, 3 dpf larval zebrafish were randomly transferred to six-well cell culture plates with a density of approximately 20 larvae per well. Then they were treated with 80 μM AlCl_3_ from 3 to 6 dpf to generate the zebrafish AD models.

### *Eucommia ulmoides* Olive Male Flower and Donepezil Treatments

**FIGURE 1:**
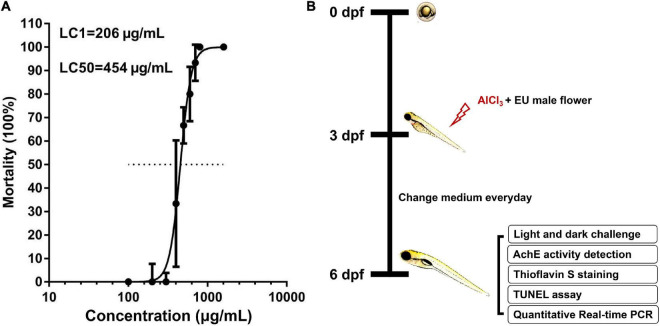
Mortality curve and experimental workflow chart. **(A)** Larval zebrafish were exposed to different concentrations of EUMF (100, 200, 300, 400, 500, 600, 700, 800, and 1,600 μg/mL) from 3 to 6 dpf. The mortality was recorded within each group at 3, 4, 5, and 6 dpf. Dead larvae were judged using missing heartbeats. **(B)** Larvae at 3 dpf were co-exposed to AlCl_3_ and three different concentrations of EUMF from 3 to 6 dpf. At 6 dpf the zebrafish were subjected to a behavioral test. In addition, we also evaluated the AchE activity, Aβ deposition, and apoptosis in the brain and performed RT-qPCR.

The larvae were treated with different concentrations of EUMF (100, 200, 300, 400, 500, 600, 700, 800, and 1,600 μg/mL) from 3 to 6 dpf. We found that the LC1 and LC50 of the EUMF were 206 and 454 μg/mL, respectively, based on the EUMF lethality curve of Figure 1A. LC_1_ is typically regarded as a no-observed-effect concentration value. Therefore, we tested the anti-AD activity of the EUMF at concentrations below LC_1_ (206 μg/mL). The zebrafish larvae were co-treated with 80 μM AlCl_3_ and EUMF at three different concentrations (50, 100, and 200 μg/mL) from 3 to 6 dpf (Figure 1B). Donepezil which is the inhibitor of acetylcholinesterase (Ache) was used as the positive drug. In the positive group, the larvae were co-treated with 80 μM AlCl_3_ and 4.0 μM donepezil from 3 to 6 dpf. After treatment, 10 larvae from each group were randomly selected for the image acquisition.

### Behavioral Analysis

The larvae from each group were randomly collected, and cleaned using an embryo medium (1 mM MgSO_4_, 0.5 mM KCl, 15 mM NaCl, 0.05 mM (NH4)_3_PO_4_, 0.15 mM KH_2_PO_4_, 0.7 mM NaHCO_3_, and 1 mM CaCl_2_). They were then placed in 48-well plates. After a 20-min acclimation period, the locomotor activity for each larva was recorded using an automated computerized video-tracking system (Viewpoint, Lyon, France). The behavioral tests contained three alternating light-dark cycles with 60 min (10 min illumination, 10 min darkness alternately). Zeblab software (Viewpoint, Lyon, France) was used to recorded and analyzed the zebrafish movement distance and speed change to light-dark and dark-light cycles.

### Detection of the Amyloid β-Protein Deposition

The zebrafish larvae were fixed using 4% paraformaldehyde. All of the fixed zebrafish were processed by embedding in the optimal cutting temperature compound (OCT Compound, SAKURA, United States) and frozen at −20°C until sectioning. Subsequently, the tissue sections were used for thioflavin S staining (Chao et al., 2018). In brief, the sections were washed with 0.01 M phosphate buffered solution (PBS) for 30 min at room temperature. Next, 0.3% thioflavin S (Sigma-Aldrich, Darmstadt, Germany) was introduced, and the sections were incubated for 8 min at room temperature in the dark. Finally, the sections were washed with 0.01 M PBS for 30 min in dark, and a fluorescence microscope (Zeiss, Jena, Germany) was used to analyze the sections. The fluorescence intensity of the Aβ deposition in the head was measured using Image-Pro Plus version 5.1.

### Determination of Ache Activity

After co-treatment with AlCl_3_ and EUMF, zebrafish larvae at 6 dpf were killed by tricaine (Sigma-Aldrich, Darmstadt, Germany). Cold physiological saline was added to the larvae in a 2 mL tube at a ratio of 1:9 (mass:volume) without any additional water. Next, the samples were homogenized using automated tissue homogenization, followed by centrifuged at 2,500 rpm for 10 min at 0°C. The supernatant was collected for the assay. The enzyme activity of Ache was determined by using the Amplite™ Fluorimetric Acetylcholinesterase Assay Kit (AAT Bioquest, California, United States) according to the manufacturer’s instructions with a slight modification as follows. The acetylthiocholine reaction mixture was 50 μM.

The test samples addition added into the acetylthiocholine reaction mixture was also 50 μM. The fluorescence at Ex/Em = 490/520 was monitored.

### Apoptosis Assessment

Apoptotic cells in the head were assessed using the One Step TUNEL Apoptosis Assay Kit (Beyotime, Jiangsu, China). Briefly, the zebrafish larvae at 6 dpf were fixed in 4% paraformaldehyde. Next, they were blocked with 3% hydrogen peroxide in methanol and incubated with the TUNEL reaction mixture. The larvae were photographed by using a fluorescence microscope (Zeiss, Jena, Germany). The fluorescence intensities of apoptotic cells in the head were measured using Image-Pro Plus version 5.1.

### Detection of Gene Expression

The expression of six genes: *autophagy and beclin 1 regulator 1a* (*ambra1a*), *autophagy-related gene 5* (*atg5*), *unc-51 like autophagy activating kinase 1* (*ulk1b*), *autophagy-related ubiquitin-like modifier LC3 B* (*lc3b*), *acetylcholinesterase* (*ache*), and *solute carrier family 6 member 3* (*slc6a3*) were detected in the zebrafish larvae using RT-qPCR. The total RNA was extracted from the larval tissue using the EASY spin Plus RNA Mini Kit (Aidlab Biotechnologies, Beijing, China) according to manufacturer instructions. Next, RNA was reverse transcribed into cDNA using the PrimeScript™ RT Master Mix (Takara Biomedical Technology Co., Ltd., Beijing, China), The RT-qPCR was conducted using the SYBR^®^ Premix DimerEraser™ (Takara Biomedical Technology Co., Ltd., Beijing, China). The housekeeping gene, *rpl13a*, was used as a reference gene. The primer sequences of the above genes are shown in Table 1.

**TABLE 1:** The sequences of primer pairs used in real-time quantitative PCR assay.

No	Gene symbol	Forward primer	Reverse primer
1	*ambra1a*	TAACCAGGAAACTGGCCAAC	AATATGCTGCAGGGGACAAC
2	*atg5*	AGGGGATAACAGCACAAACG	CTTCTTATGCAGCGTGTCCA
3	*ulk1b*	AGGCCGAAAGTCTCACTTCA	AGCCATGTACATCGGAGACC
4	*lc3b*	CCTCCAACTCAACTCCAACC	GCCGTCTTCGTCTCTTTCC
5	*ache*	TCTTGCCCACTGTGCTACTC	TCTTGTACCCTGCACTCTGC
6	*slc6a3*	CTAATCGCCTTCTCCAGCTACA	GGCCACGTTGTGTTTCTGTGACAT
7	*rpl13a*	TCTGGAGGACTGTAAGAGGTATGC	AGACGCACAATCTTGAGAGCAG

### Statistical Analysis

The data are presented as mean ± SEM. The statistical analyses were conducted using Graph Pad Prism 8.0 (GraphPad Software; San Diego, CA, United States) by a one-way ANOVA followed by the Dunnett’s multiple comparison test. If the *P*-value was less than 0.05, the difference was considered as significant.

## Results

### Flavonoids Compounds Analysis of the *Eucommia ulmoides* Olive Male Flower

A large number of literature studies have shown that flavonoids show neuroprotective effects against AD (Remya et al., 2012; Remya et al., 2014; Bakhtiari et al., 2017; Zhao et al., 2019; Li et al., 2021; Noori et al., 2021; Pragya and Arun, 2021). Thus, the flavonoids were selected as the primary components of EUMF for further study. The total contents of the EUMF flavonoids were determined according to the obtained standard curves of the total flavonoids which was reported in a previous study from our lab (Zhang et al., 2020). According to regression equations (*y* = 0.0003x + 0.0107), the total contents of the EUMF flavonoids were 45.99 ± 0.5853 mg/g. Moreover, the targeted LC-MS analysis of the flavonoids showed that total 206 compounds were detected (Table 2). Among them, 125 flavonoids and flavanols, 32 flavanones, 22 isoflavonoids, 11 chalcones and dihydrochalcones, and 17 anthocyanins were identified. In addition, quercetin-3′-O-glucoside (relative content of 9.1850%), isoquercitrin (8.8025%), rutin hydrate (8.0753%), rutin (7.9072%), spiraeoside (7.5218%), isotrifoliin (7.3683%), isorhamnetin-3-O-neohespeidoside (6.5757%), naringenin (5.7225%), naringenin chalcone (5.6733%), butin, myricitrin, isomucronulatol-7-O-glucoside, hyperoside, hesperetin 5-O-glucoside, narcissoside, di-O-methylquercetin, lonicerin and morin were the primary components of EUMF. The relative content of these 18 components accounted for greater than 90% of the total flavonoids.

**TABLE 2:** Flavonoids compounds identified in EUMF by LC-MS.

No	RT (min)	Molecular Weight	Formula	Name	Relative content (%)	Class
1	0.680	274.084	C_15_H_14_O_5_	Afzelechin	0.0854	Flavonoids
2	0.700	420.454	C_25_H_24_O_6_	Kuwanon A	0.0064	Flavones and Flavanols
3	0.710	448.400	C_22_H_22_O_11_	Methylluteolin C-hexoside	0.0210	Flavones and Flavanols
4	0.720	418.100	C_21_H_22_O_9_	O-methylnaringenin C-pentoside	0.2106	Flavanones
5	0.720	418.394	C_21_H_22_O_9_	Methylnaringenin C-pentoside	0.2024	Flavanones
6	0.730	402.350	C_20_H_18_O_9_	Apigenin C-pentoside	0.0411	Flavones and Flavanols
7	0.730	446.404	C_22_H_22_O_10_	Methylapigenin C-hexoside	0.0837	Flavones and Flavanols
8	0.730	550.460	C_25_H_26_O_14_	di-C, C-pentosyl-luteolin	0.0554	Flavones and Flavanols
9	0.740	478.400	C_22_H_22_O_12_	Selgin C-hexoside	0.0150	Flavones and Flavanols
10	0.780	342.343	C_19_H_18_O_6_	Methylophiopogonanone A	0.0025	Isoflavonoids
11	0.940	478.400	C_22_H_22_O_12_	Selgin 5-O-hexoside	0.0348	Flavones and Flavanols
12	0.950	272.069	C_15_H_12_O_5_	Butein	0.0093	Chalcones and dihydrochalcones
13	0.970	476.430	C_23_H_24_O_11_	Irisolidone 7-O-beta-d-glucoside	0.0106	Isoflavonoids
14	0.970	476.430	C_23_H_24_O_11_	Methylchrysoeriol 5-O-hexoside	0.0136	Flavones and Flavanols
15	0.970	508.430	C_23_H_24_O_13_	Limocitrin O-hexoside	0.0236	Flavones and Flavanols
16	0.980	756.660	C_33_H_40_O_20_	C-hexosyl-apigenin O-hexosyl-O-hexoside	0.0048	Flavones and Flavanols
17	0.980	479.000	C_22_H_23_O_12_	Petunidin 3-O-glucoside	0.0489	Anthocyanins
18	1.000	624.552	C_28_H_32_O_16_	C-hexosyl-chrysoeriol O-hexoside	0.0105	Flavones and Flavanols
19	1.010	286.279	C_16_H_14_O_5_	Sakuranetin	0.0118	Flavanones
20	1.010	416.378	C_21_H_20_O_9_	Methylapigenin C-pentoside	0.0043	Flavones and Flavanols
21	1.040	430.405	C_22_H_22_O_9_	Ononin	0.0468	Isoflavonoids
22	1.130	868.702	C_43_H_32_O_20_	8-Gingerol	0.0069	Flavonoids
23	1.220	576.500	C_30_H_24_O_12_	Procyanidin A1	0.0009	Anthocyanins
24	1.260	576.500	C_30_H_24_O_12_	Procyanidin A2	0.0011	Anthocyanins
25	1.284	254.240	C_15_H_10_O_4_	Chrysin	0.0004	Flavones and Flavanols
26	2.600	332.262	C_16_H_12_O_8_	Laricitrin	0.4216	Flavones and Flavanols
27	4.440	302.279	C_16_H_14_O_6_	Homoeriodictyol	0.0003	Flavanones
28	4.560	302.043	C_15_H_10_O_7_	Tricetin	0.0003	Flavones and Flavanols
29	5.098	306.270	C_15_H_14_O_7_	(-)-epigallocatechin	0.0005	Flavonoids
30	5.210	484.840	C_21_H_21_ClO_11_	Cyanidin 3-O-glucoside	0.0111	Anthocyanins
31	5.213	484.840	C_21_H_21_ClO_11_	Idaein chloride	0.0081	Anthocyanins
32	5.310	466.392	C_21_H_22_O_12_	Taxifolin O-glucoside	0.0064	Flavanones
33	5.380	356.332	C_19_H_16_O_7_	Ophiopogonanone C	0.2663	Flavanones
34	5.390	626.520	C_27_H_30_O_17_	Quercetin-3,4′-O-di-beta-glucopyranoside	0.3593	Flavones and Flavanols
35	5.420	809.120	C_33_H_41_O_21_C_*l1*_	Delphinidin 3-sophoroside-5-rhamnoside	0.0329	Anthocyanins
36	5.442	468.840	C_21_H_21_ClO_10_	Callistephin chloride	0.0005	Anthocyanins
37	5.515	528.890	C_23_H_25_ClO_12_	Malvidin 3-galactoside chloride	0.0005	Anthocyanins
38	5.572	528.890	C_23_H_25_ClO_12_	Oenin chloride	0.0022	Anthocyanins
39	5.602	338.700	C_15_H_11_ClO_7_	Delphinidin chloride	0.0009	Anthocyanins
40	5.680	610.518	C_27_H_30_O_16_	C-hexosyl-luteolin O-hexoside	0.0012	Flavones and Flavanols
41	5.730	594.518	C_27_H_30_O_15_	Apigenin-6,8-di-C-glycoside	0.1253	Flavones and Flavanols
42	5.745	432.380	C_21_H_20_O_10_	Puerarin	0.0031	Isoflavonoids
43	5.810	624.544	C_28_H_32_O_16_	di-C,C-hexosyl-methylluteolin	0.1441	Flavones and Flavanols
44	5.820	594.518	C_27_H_30_O_15_	di-C,C-hexosyl-apigenin	0.2993	Flavones and Flavanols
45	5.890	288.252	C_15_H_12_O_6_	Fustin	0.0043	Flavanones
46	6.030	564.499	C_26_H_28_O_14_	Isoschaftoside	0.0142	Flavones and Flavanols
47	6.040	594.526	C_27_H_30_O_15_	C-pentosyl-chrysoeriol O-hexoside	0.0236	Flavones and Flavanols
48	6.050	564.490	C_26_H_28_O_14_	C-pentosyl-C-hexosyl-apigenin	0.0028	Flavones and Flavanols
49	6.074	564.492	C_26_H_28_O_14_	Schaftoside	0.0828	Flavones and Flavanols
50	6.080	430.628	C_27_H_42_O_4_	Hecogenin	0.0367	Flavonoids
51	6.090	466.398	C_21_H_22_O_12_	Plantagoside	0.0256	Flavanones
52	6.100	448.400	C_21_H_20_O_11_	Luteolin C-hexoside derivative	0.2109	Flavones and Flavanols
53	6.174	448.380	C_21_H_20_O_11_	Isoorientin	0.0972	Flavones and Flavanols
54	6.230	416.378	C_21_H_20_O_9_	Toringin	0.0272	Flavones and Flavanols
55	6.230	446.121	C_22_H_22_O_10_	Sissotrin	0.0007	Isoflavonoids
56	6.250	448.377	C_21_H_20_O_11_	Orientin	0.0972	Flavones and Flavanols
57	6.263	322.700	C_15_H_11_ClO_6_	Cyanidin chloride	0.0007	Anthocyanins
58	6.270	594.518	C_27_H_30_O_15_	Saponarin	0.0360	Flavones and Flavanols
59	6.280	610.525	C_27_H_30_O_16_	Kaempferol-3-gentiobioside	0.0200	Flavones and Flavanols
60	6.280	594.518	C_27_H_30_O_15_	4′-O-Glucosylvitexin	0.0360	Flavones and Flavanols
61	6.300	578.519	C_27_H_30_O_14_	6”-O-xylosyl-glycitin	0.0023	Isoflavonoids
62	6.350	611.500	C_27_H_31_O_16_	Tulipanin	0.3871	Anthocyanins
63	6.360	610.520	C_27_H_30_O_16_.xH_2_O	Rutin hydrate	8.0753	Flavones and Flavanols
64	6.380	596.542	C_27_H_32_O_15_	Neoeriocitrin	0.4294	Flavanones
65	6.390	434.121	C_21_H_22_O_10_	Isohemiphloin	0.0171	Flavanones
66	6.411	578.520	C_27_H_30_O_14_	Vitexin-2-O-rhaMnoside	0.0026	Flavones and Flavanols
67	6.430	612.576	C_28_H_36_O_15_	Neohesperidin dihydrochalcone	0.1784	Chalcones and dihydrochalcones
68	6.430	596.534	C_27_H_32_O_15_	Eriocitrin	0.0007	Flavanones
69	6.451	610.518	C_27_H_30_O_16_	Rutin	7.9072	Flavones and Flavanols
70	6.460	432.113	C_21_H_20_O_10_	Apigenin C-glucoside	0.0009	Flavones and Flavanols
71	6.470	208.255	C_15_H_12_O	Chalcone	0.5983	Chalcones and dihydrochalcones
72	6.510	594.526	C_27_H_30_O_15_	Kaempferol-3-O-rutinoside	0.0309	Flavones and Flavanols
73	6.530	462.366	C_21_H_18_O_12_	Luteolin-7-O-beta-D-glucuronide	0.0017	Flavones and Flavanols
74	6.530	464.382	C_21_H_20_O_12_	Quercetin-3′-O-glucoside	9.1850	Flavones and Flavanols
75	6.530	464.469	C_23_H_28_O_10_	Isomucronulatol-7-O-glucoside	3.3727	Isoflavonoids
76	6.533	432.378	C_21_H_20_O_10_	Isovitexin	0.0007	Flavones and Flavanols
77	6.540	464.376	C_21_H_20_O_12_	Quercetin-O-glucoside	0.4630	Flavones and Flavanols
78	6.547	464.380	C_21_H_20_O_12_	Myricitrin	4.7113	Flavones and Flavanols
79	6.550	594.159	C_27_H_30_O_15_	Kaempferol 3-O-robinobioside	0.4242	Flavones and Flavanols
80	6.554	464.380	C_21_H_20_O_12_	Hyperoside	2.9062	Flavones and Flavanols
81	6.559	432.380	C_21_H_20_O_10_	Vitexin	0.0006	Flavones and Flavanols
82	6.581	446.404	C_22_H_22_O_10_	Calycosin-7-O-beta-D-glucoside	0.0027	Isoflavonoids
83	6.590	464.419	C_22_H_24_O_11_	Hesperetin 5-O-glucoside	1.6133	Flavanones
84	6.590	464.096	C_21_H_20_O_12_	Spiraeoside	7.5218	Flavones and Flavanols
85	6.590	464.096	C_21_H_20_O_12_	Isotrifoliin	7.3683	Flavones and Flavanols
86	6.590	462.360	C_21_H_18_O_12_	Scutellarin	0.0010	Flavones and Flavanols
87	6.596	464.380	C_21_H_20_O_12_	Isoquercitrin	8.8025	Flavones and Flavanols
88	6.620	448.101	C_21_H_20_O_11_	Trifolin	0.1696	Flavones and Flavanols
89	6.620	448.383	C_21_H_20_O_11_	Luteoloside	0.1774	Flavones and Flavanols
90	6.650	448.377	C_21_H_20_O_11_	Kaempferol7-O-beta-D-glucopyranoside	0.1172	Flavones and Flavanols
91	6.664	448.380	C_21_H_20_O_11_	Luteolin 7-O-glucoside	0.1331	Flavones and Flavanols
92	6.680	432.380	C_21_H_20_O_10_	Apigenin 5-O-glucoside	0.0666	Flavones and Flavanols
93	6.720	462.404	C_22_H_22_O_11_	Chrysoeriol C-hexoside	0.0445	Flavones and Flavanols
94	6.730	594.526	C_27_H_30_O_15_	Lonicerin	1.4742	Flavones and Flavanols
95	6.780	625.560	C_28_H_33_O_16_	Petunidin 3-O-rutinoside	0.0030	Anthocyanins
96	6.780	432.380	C_21_H_20_O_10_	Genistin	0.0018	Isoflavonoids
97	6.790	624.552	C_28_H_32_O_16_	Isorhamnetin-3-O-neohespeidoside	6.5757	Flavones and Flavanols
98	6.825	624.544	C_28_H_32_O_16_	Narcissoside	1.5707	Flavones and Flavanols
99	6.837	580.535	C_27_H_32_O_14_	Narirutin	0.0171	Flavanones
100	6.840	578.520	C_27_H_30_O_14_	Isorhoifolin	0.1097	Flavones and Flavanols
101	6.860	462.410	C_22_H_22_O_11_	Pratensein-7-O-glucoside	0.1006	Isoflavonoids
102	6.864	462.400	C_22_H_22_O_11_	Tectoridin	0.0013	Isoflavonoids
103	6.880	316.262	C_16_H_12_O_7_	Rhamnetin	0.0394	Flavones and Flavanols
104	6.885	304.250	C_15_H_12_O_7_	Taxifolin	0.2520	Flavones and Flavanols
105	6.900	268.269	C_16_H_12_O_4_	Tectochrysin	0.0005	Flavones and Flavanols
106	6.910	306.700	C_15_H_11_ClO_5_	Pelargonidin chloride	0.0008	Anthocyanins
107	6.940	608.545	C_28_H_32_O_15_	Chrysoeriol 7-O-rutinoside	0.0021	Flavones and Flavanols
108	6.940	578.520	C_27_H_30_O_14_	Rhoifolin	0.2416	Flavones and Flavanols
109	6.970	270.241	C_15_H_10_O_5_	6,7,4′-Trihydroxyisoflavone	0.0012	Isoflavonoids
110	6.970	448.383	C_21_H_20_O_11_	Vincetoxicoside B	0.0033	Flavones and Flavanols
111	6.979	580.530	C_27_H_32_O_14_	Naringin	0.0110	Flavanones
112	6.980	418.390	C_21_H_22_O_9_	Liquiritin	0.0054	Flavanones
113	7.010	502.200	C_26_H_30_O_10_	Phellodensin F	0.0034	Flavanones
114	7.020	434.121	C_21_H_22_O_10_	Prunin	0.0868	Flavanones
115	7.038	432.380	C_21_H_20_O_10_	Sophoricoside	0.0075	Isoflavonoids
116	7.060	272.069	C_15_H_12_O_5_	Pinobanksin	0.1659	Flavanones
117	7.080	446.367	C_21_H_18_O_11_	Apigenin7-O-beta-D-glucuronide	0.0006	Flavones and Flavanols
118	7.080	418.351	C_20_H_18_O_10_	Kaempferol 3-A-L-Arabinopyranoside	0.0060	Flavones and Flavanols
119	7.080	432.420	C_22_H_24_O_9_	Heptamethoxyflavone	0.0076	Flavones and Flavanols
120	7.080	434.400	C_21_H_20_O_10_	Resokaempferol 7-O-hexoside	0.0077	Flavones and Flavanols
121	7.120	608.545	C_28_H_32_O_15_	Neodiosmin	0.0006	Flavones and Flavanols
122	7.120	536.000	C_24_H_24_O_14_	Eriodictyol O-malonylhexoside	0.0030	Flavanones
123	7.180	462.404	C_22_H_22_O_11_	Chrysoeriol 5-O-hexoside	0.0047	Flavones and Flavanols
124	7.186	462.403	C_22_H_22_O_11_	Homoplantaginin	0.0058	Flavones and Flavanols
125	7.190	480.376	C_21_H_20_O_13_	Myricetin 3-O-galactoside	0.0185	Flavones and Flavanols
126	7.190	492.430	C_23_H_24_O_12_	Tricin 5-O-hexoside	0.0017	Flavones and Flavanols
127	7.200	610.560	C_28_H_34_O_15_	Neohesperidin	0.0461	Flavanones
128	7.200	448.400	C_22_H_22_O_11_	Chrysoeriol 7-O-hexoside	0.0087	Flavones and Flavanols
129	7.240	448.380	C_21_H_20_O_11_	Quercitrin	0.0074	Flavones and Flavanols
130	7.240	436.410	C_21_H_24_O_10_	Phlorizin	0.0718	Chalcones and dihydrochalcones
131	7.300	476.430	C_23_H_24_O_11_	Methylchrysoeriol C-hexoside	0.0030	Flavones and Flavanols
132	7.320	526.490	C_27_H_26_O_11_	Tricin 4′-O-(beta-guaiacylglyceryl) ether	0.0316	Flavones and Flavanols
133	7.340	318.240	C_15_H_10_O_8_	Myricetin	0.0088	Flavones and Flavanols
134	7.350	432.106	C_21_H_20_O_10_	Kaempferin	0.0009	Flavones and Flavanols
135	7.360	432.106	C_21_H_20_O_10_	Kaempferol 7-O-rhamnoside	0.0007	Flavones and Flavanols
136	7.372	582.550	C_27_H_34_O_14_	Naringin dihydrochalcone	0.0001	Flavanones
137	7.400	688.639	C_33_H_36_O_16_	Tricin 4′-O-(β-guaiacylglyceryl) ether O-hexoside	0.0165	Flavones and Flavanols
138	7.417	286.240	C_15_H_10_O_6_	Fisetin	0.0190	Flavones and Flavanols
139	7.471	418.394	C_21_H_22_O_9_	Isoliquiritin	0.0068	Chalcones and dihydrochalcones
140	7.500	330.289	C_17_H_14_O_7_	Tricin	0.1054	Flavones and Flavanols
141	7.500	578.470	C_26_H_26_O_15_	Tricin O-malonylhexoside	0.0295	Flavones and Flavanols
142	7.510	688.630	C_33_H_36_O_16_	Tricin 4′-O-(beta-guaiacylglyceryl) ether 5-O-hexoside	0.0137	Flavones and Flavanols
143	7.523	436.409	C_21_H_24_O_10_	Trilobatin	0.0356	Chalcones and dihydrochalcones
144	7.575	446.360	C_21_H_18_O_11_	Baicalin	0.0095	Flavones and Flavanols
145	7.635	286.236	C_15_H_10_O_6_	Scutellarein	0.0022	Flavones and Flavanols
146	7.660	314.289	C_17_H_14_O_6_	Kumatakenin	0.0134	Flavones and Flavanols
147	7.660	254.240	C_15_H_10_O_4_	4′,7-Dihydroxyflavone	0.0164	Flavones and Flavanols
148	7.670	534.420	C_24_H_22_O_14_	Tricin 5-O-hexoside derivative	0.0073	Flavones and Flavanols
149	7.790	592.553	C_28_H_32_O_14_	Linarin	0.0019	Flavones and Flavanols
150	7.860	622.571	C_29_H_34_O_15_	Pectolinarin	0.0015	Flavones and Flavanols
151	7.879	594.520	C_30_H_26_O_13_	Tiliroside	0.0020	Flavones and Flavanols
152	7.971	416.000	C_21_H_20_O_9_	Apigenin 4-O-rhamnoside	0.0002	Flavones and Flavanols
153	7.999	288.252	C_15_H_12_O_6_	Eriodictyol	0.3773	Flavanones
154	8.020	286.048	C_15_H_10_O_6_	2′-Hydroxygenistein	0.0155	Isoflavonoids
155	8.049	594.561	C_28_H_34_O_14_	Poncirin	0.0011	Flavanones
156	8.050	302.043	C_15_H_10_O_7_	Morin	1.3804	Flavones and Flavanols
157	8.060	286.240	C_15_H_10_O_6_	Luteolin	0.0010	Flavones and Flavanols
158	8.100	668.600	C_33_H_32_O_15_	Tricin O-sinapoylpentoside	0.0003	Flavones and Flavanols
159	8.180	284.263	C_16_H_12_O_5_	Calycosin	0.0329	Isoflavonoids
160	8.229	314.246	C_16_H_10_O_7_	Wedelolactone	0.0004	Isoflavonoids
161	8.260	460.388	C_22_H_20_O_11_	Wogonoside	0.0027	Flavones and Flavanols
162	8.518	272.253	C_15_H_12_O_5_	Naringenin chalcone	5.6733	Chalcones and dihydrochalcones
163	8.519	500.840	C_21_H_21_ClO_12_	Myrtillin chloride	0.0002	Anthocyanins
164	8.598	274.270	C_15_H_14_O_5_	Phloretin	0.2532	Chalcones and dihydrochalcones
165	8.610	272.069	C_15_H_12_O_5_	Butin	4.9779	Flavanones
166	8.645	270.280	C_16_H_14_O_4_	Echinatin	0.0001	Chalcones and dihydrochalcones
167	8.683	272.250	C_15_H_12_O_5_	Naringenin	5.7225	Flavanones
168	8.700	270.240	C_15_H_10_O_5_	Apigenin	0.0057	Flavones and Flavanols
169	8.720	270.240	C_15_H_10_O_5_	Genistein	0.0002	Isoflavonoids
170	8.800	302.327	C_17_H_18_O_5_	Isomucronulatol	0.0010	Isoflavonoids
171	8.811	286.240	C_15_H_10_O_6_	Kaempferol	0.0106	Flavones and Flavanols
172	8.849	300.263	C_16_H_12_O_6_	Tectorigenin	0.0009	Isoflavonoids
173	8.860	302.079	C_16_H_14_O_6_	7-O-Methyleriodictyol	0.0017	Flavanones
174	8.911	300.260	C_16_H_12_O_6_	Diosmetin	0.0031	Flavones and Flavanols
175	8.960	302.236	C_15_H_10_O_7_	Quercetin	0.0197	Flavones and Flavanols
176	8.969	316.262	C_16_H_12_O_7_	Isorhamnetin	0.0097	Flavones and Flavanols
177	8.972	302.270	C_16_H_14_O_6_	Hesperetin	0.0073	Flavanones
178	9.020	330.074	C_17_H_14_O_7_	3,7-Di-O-methylquercetin	0.0008	Flavones and Flavanols
179	9.160	360.320	C_18_H_16_O_8_	5,7,3′-trihydroxy-6,4′,5′-trimethoxyflavone	0.0000	Flavones and Flavanols
180	9.190	330.100	C_17_H_14_O_7_	Di-O-methylquercetin	1.5690	Flavones and Flavanols
181	9.370	372.375	C_20_H_20_O_7_	Isosinensetin	0.0001	Flavones and Flavanols
182	9.550	372.370	C_20_H_20_O_7_	Sinensetin	0.0002	Flavones and Flavanols
183	9.590	338.360	C_20_H_18_O_5_	Wighteone	0.0004	Isoflavonoids
184	9.594	300.263	C_16_H_12_O_6_	Hydroxygenkwanin	0.6367	Flavones and Flavanols
185	9.870	284.263	C_16_H_12_O_5_	Maackiain	0.0168	Isoflavonoids
186	9.935	300.310	C_17_H_16_O_5_	Farrerol	0.0002	Flavanones
187	10.004	344.320	C_18_H_16_O_7_	Eupatilin	0.0001	Flavones and Flavanols
188	10.110	284.225	C_15_H_8_O_6_	Rhein	0.0004	Anthocyanins
189	10.287	284.260	C_16_H_12O5_	Wogonin	0.0014	Flavones and Flavanols
190	10.315	286.279	C_16_H_14_O_5_	Isosakuranetin	0.0023	Flavanones
191	10.340	374.347	C_19_H_18_O_8_	Chrysosplenetin B	0.0001	Flavones and Flavanols
192	10.372	256.250	C_15_H_12_O_4_	Pinocembrin	0.0015	Flavanones
193	10.373	514.520	C_27_H_30_O_10_	Baohuoside I	0.0004	Flavones and Flavanols
194	10.374	374.341	C_19_H_18_O_8_	Casticin	0.0005	Flavones and Flavanols
195	10.400	286.328	C_17_H_18_O_4_	Loureirin A	0.0010	Chalcones and dihydrochalcones
196	10.518	402.390	C_21_H_22_O_8_	Nobiletin	0.0046	Flavones and Flavanols
197	10.520	356.332	C_19_H_16_O_7_	6-Formyl-isoophiopogonanone A	0.0022	Flavanones
198	10.550	284.263	C_16_H_12_O_5_	Oroxylin A	0.0005	Flavones and Flavanols
199	10.780	314.295	C_17_H_14_O_6_	Pectolinarigenin	0.0002	Flavones and Flavanols
200	11.410	372.370	C_20_H_20_O_7_	Tangeretin	0.0030	Flavones and Flavanols
201	11.419	336.720	C_16_H_13_ClO_6_	Peonidin chloride	0.0007	Anthocyanins
202	11.520	388.368	C_20_H_20_O_8_	Demethylnobiletin	0.0001	Flavones and Flavanols
203	11.564	224.250	C_15_H_12_O_2_	Flavanone	0.0007	Flavanones
204	11.740	298.295	C_17_H_14_O_5_	Mosloflavone	0.0009	Flavones and Flavanols
205	11.930	324.370	C_20_H_20_O_4_	Isobavachalcone	0.0005	Chalcones and dihydrochalcones
206	12.143	394.420	C_23_H_22_O_6_	Deguelin	0.0001	Isoflavonoids
207	12.670	368.380	C_21_H_20_O_6_	Anhydroicaritin	0.0000	Flavones and Flavanols

### Dyskinesia Rehabilitation Effects of *Eucommia ulmoides* Olive Male Flower in Zebrafish Larvae

**FIGURE 2:**
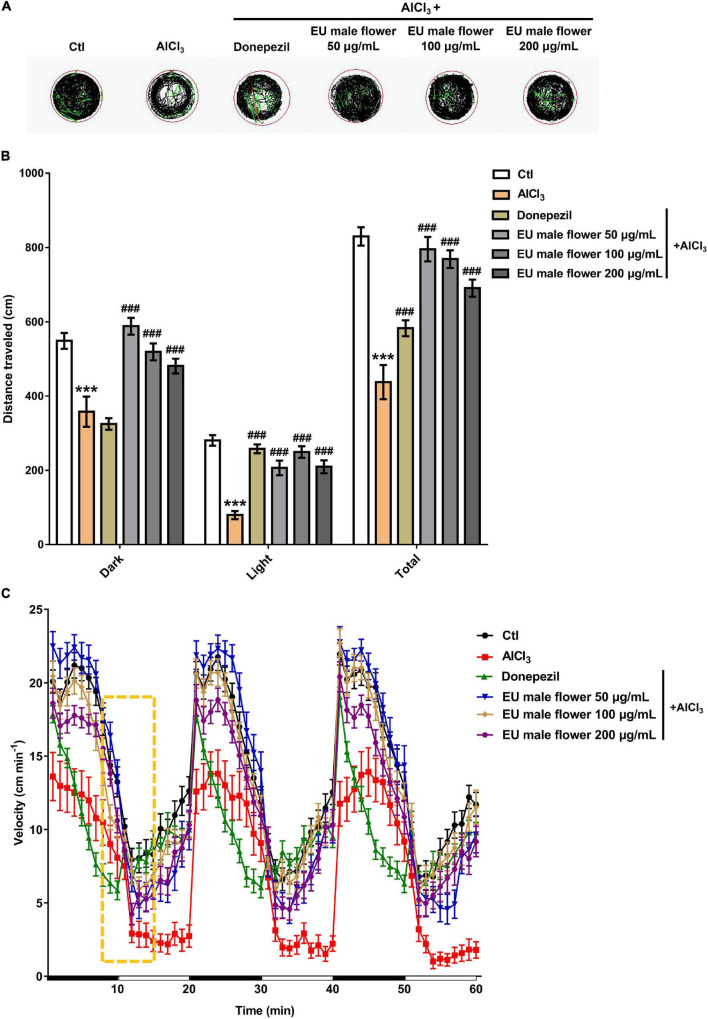
Effect of EUMF on AlCl_3_-induced locomotion impairments in zebrafish. **(A)** Dgital track map. The red, green, and black lines depict fast, medium, and slow movements, respectively (*n* = 10). **(B)** Total distance moved in the Ctl, AlCl_3_, and AlCl_3_ + EUMF groups (***P < 0.001 vs. Ctl; ^###^*P* < 0.001 vs. AlCl_3_; *n* = 10). **(C)** Speed change in the Ctl, AlCl_3_, and AlCl_3_ + EUMF groups (the speed change after light stimulus is demarcated by the frame; *n* = 10).

Behavioral tests were performed on the zebrafish larvae at 6 dpf. As shown in Figure 2A, The black lines, green lines, and red lines indicate slow, medium, and fast movements, respectively. We found that the distance traveled by zebrafish in the AD model group was significantly shorter than the zebrafish in the untreated group, whether in light or dark environments (Figure 2B). The speed change of the zebrafish in the AD model group was also notably weakened after light stimulus alteration compared with the zebrafish in the untreated group (Figure 2C). These results indicated that AlCl_3_ lessened the locomotor capacity of the zebrafish, and this was consistent with the previous study (Pan et al., 2019; Li et al., 2020). Accordingly, the establishment of zebrafish AD model was successful. After treatment with 4.0 μM donepezil, the distance traveled and speed change of the zebrafish both increased compared with the zebrafish in the AD group (Figures 2B,C). This implied that donepezil improved the dyskinesia of zebrafish induced by AlCl_3_. Interestingly, a similar trend of behavioral change in the positive group was also observed in the EUMF treatment groups. When the zebrafish were co-treated with AlCl_3_ and different concentrations of the EUMF (50, 100, and 200 μg/mL), their dyskinesias were also reduced. In particular, the EUMF treatment correlated with a longer distance in dark environments than that of the donepezil group (Figures 2B,C). The above results indicated that the EUMF improved the exercise capacity and may play a protective role against AlCl_3_-induced AD-like symptoms in zebrafish.

### Inhibition the Amyloid β-Protein Aggregation Effects of *Eucommia ulmoides* Olive Male Flower in the Zebrafish Larvae

**FIGURE 3:**
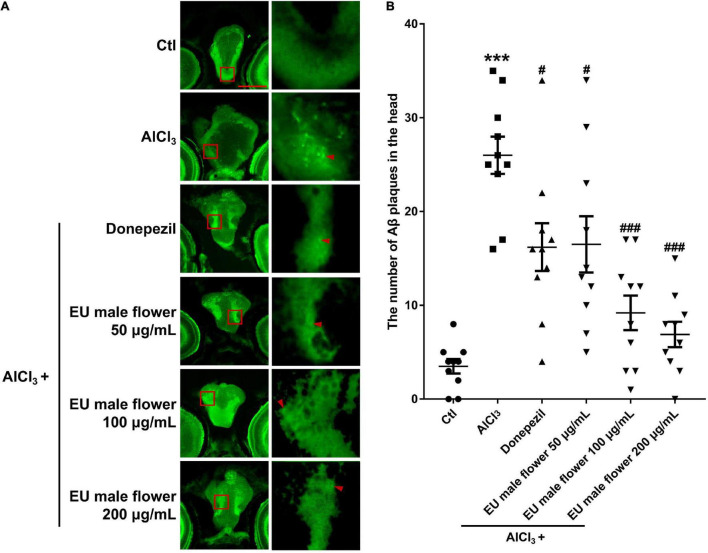
Inhibition of EUMF on Aβ aggregation in zebrafish. **(A)** The Aβ plaques in the brain region were stained using thioflavin S in the Ctl, AlCl_3_, and AlCl_3_ + EUMF groups (Aβ is demarcated by arrows; scale bar = 100 μm). **(B)** Statistical analysis of the Aβ plaque count in each group (***P < 0.001 vs. Ctl; ^#^*P* < 0.05; ^###^*P* < 0.001 vs. AlCl_3_; *n* = 10).

Amyloid β-protein deposition is an important clinical hallmark in AD patients (Meldolesi, 2017). To further identify the anti-AD activity of the EUMF, the Aβ plaques in the heads of zebrafish were quantitatively determined. As shown in Figure 3, only a few of the Aβ plaques were observed in the brain of the untreated group. In contrast, there were many large Aβ plaques in the brain of the AD model group. Compared with the AD group, larval treatment with donepezil or EUMF (50, 100, and 200 μg/mL) significantly reduced the Aβ plaque count. These results implied that EUMF had anti-AD activity.

### Inhibitory Activity of *Eucommia ulmoides* Olive Male Flower on the Ache Activity

**FIGURE 4:**
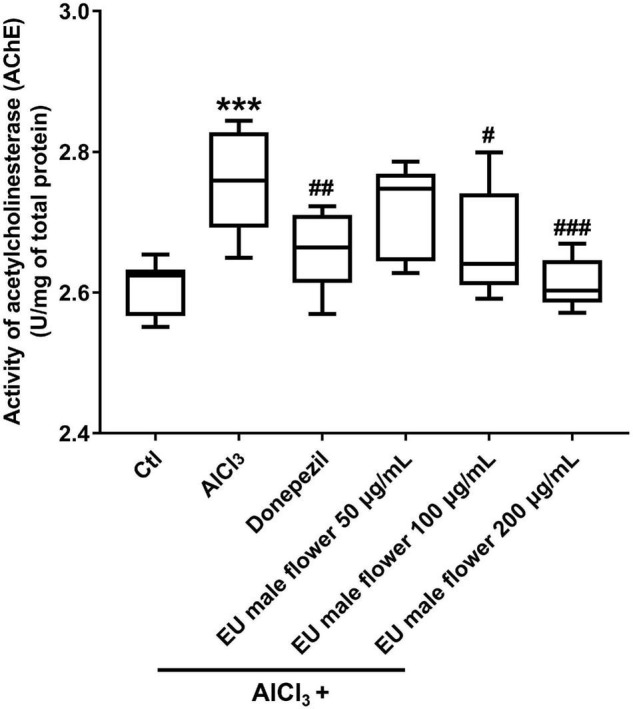
Inhibition of EUMF on the AChE activity in zebrafish (***P < 0.001 vs. Ctl; ^#^*P* < 0.05, ^##^*P* < 0.01, ^###^*P* < 0.001 vs. AlCl_3_; *n* = 10).

Ache is an enzyme that can degrade ACh. Many studies have proposed that a reduced level of ACh may be the primary etiology of AD. Hence, Ache has also been proposed to be related to the formation of AD (Remya et al., 2013; Hu et al., 2019). Based on this, we assessed the activity of Ache to explore the protective mechanism of EUMF on AD. As shown in Figure 4, the AD model group showed a higher activity of Ache compared with the untreated group. However, the groups co-treated with both AlCl_3_ and donepezil or different concentrations of EUMF showed reduced activity of Ache compared with the AD model group. Our results indicated that the EUMF may be an effective therapeutic agent for AD by suppressing the activity of Ache.

### Effect of *Eucommia ulmoides* Olive Male Flower on AlCl_3_-Induced Apoptosis in the Brain

**FIGURE 5:**
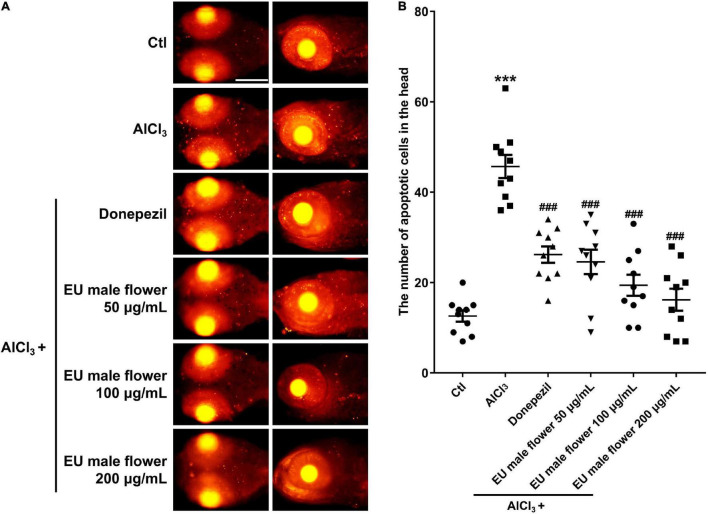
Effect of EUMF on apoptosis in the brains of the AlCl_3_-modeled zebrafish. **(A)** The apoptotic cells were stained with TUNEL (scale bar = 100 μm). **(B)** Statistical analysis of the apoptotic cells count in the larvae heads (***P < 0.001 vs. Ctl; ^###^*P* < 0.001 vs. AlCl_3_; *n* = 10).

We found many apoptotic cells that appeared primarily in the brain region in the zebrafish AD model. In contrast, no obvious apoptotic cells were observed in the control group. Donepezil or different concentrations of the EUMF treatment significantly reduced the number of apoptotic cells in the zebrafish brains (Figure 5). The above results suggested that EUMF suppressed the apoptosis induced by AlCl_3_ in zebrafish brain.

### Effect of *Eucommia ulmoides* Olive Male Flower on the Expression of Autophagy-Related and Neurotransmitter-Related Genes

**FIGURE 6:**
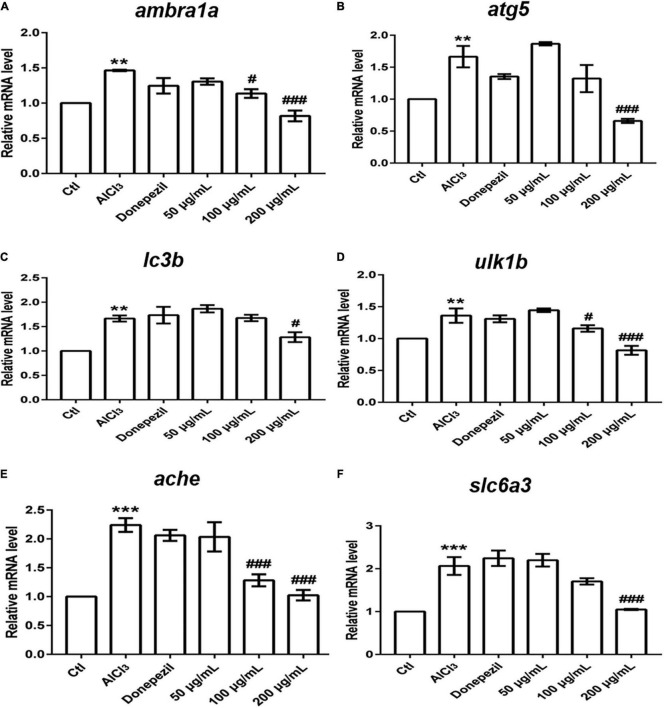
Transcriptional alterations of genes. The amount of gene expression was exhibited as the relative expression (shown as fold) compared with the Ctl. (**P < 0.01, ***P < 0.001 vs. Ctl; ^#^*P* < 0.05, ^###^*P* < 0.001 vs. AlCl_3_). **(A–D)** Expressions of genes involved in autophagy. **(E)** Transcript levels of *ache*. **(F)** Transcript levels of *slc6a3*.

Many lines of evidence have suggested that dysregulated autophagy is implicated in a pathogenic role in the neurological diseases (Sheng et al., 2010; Chu, 2018). Therefore, we assayed the expression of autophagy-related genes to investigate whether EUMF protected against AD-like symptoms by regulating autophagy. *Ambra1a*, *atg5*, *ulk1b*, and *lc3b* are core members involved in autophagy (Kang et al., 2011; Jiang et al., 2013). We found that transcript levels of aforementioned genes were significantly upregulated in the AD model group compared with the control, while when the EUMF reached a certain concentration, it reversed the increases (Figure 6). In addition, we also found that the EUMF treatment under a certain concentration downregulated the expression level of *ache* and *slc6a3*, and these were drastically increased after treatment with AlCl_3_ (Figure 6).

## Discussion

Alzheimer’s disease is the most common clinical degenerative disease associated with aging. The complex pathogenetic factors of AD have limited its effective treatment. EUO is a traditional Chinese medicine. It has been reported that the extracts of EUO leaf can be used to treat AD. Therefore, we investigated the therapeutic effects of its male flowers on AD like symptoms using zebrafish. We found that the dyskinesia in the zebrafish AD model was significantly improved by EUMF. The Aβ plaques count, Ache activity, and number of apoptotic cells in the zebrafish AD model were also clearly reduced by EUMF. The above results indicated that the EUMF may be an agent for AD treatment. In addition, mechanism investigation revealed that the anti-AD activity of the EUMF may be related to its inhibition of excessive autophagy and abnormal expressions of *ache* and *slc6a3* genes.

Autophagy is an important biological process by which cellular material is degraded by lysosomes or vacuoles and recycled. Paradoxically, it has the characteristics of a double-edged sword. Autophagy can serve to protect the nervous system by clearing degrading damaged organelles or accumulated misfolded proteins in neurons, but it may also induce neuron death and damage the nervous system (Shintani and Klionsky, 2004). Previous studies have shown that autophagy influences the secretion of Aβ to the extracellular space in neurons through either excretory or exocytic mechanisms, and hence it plays a critical role in Aβ plaque formation. Furthermore, extracellular Aβ plaques accumulation is an important pathogenic factor leading to AD (Nilsson et al., 2015). Based on these facts, we hypothesized that AlCl_3_ may activate abnormal excessive autophagy by upregulating the expression of *ambra1a*, *atg5*, *ulk1b*, and *lc3b* in zebrafish. Then further damage, referring to the deposition of extracellular Aβ plaques induced by abnormal excessive autophagy, would occur. Finally, AD-like symptoms in the zebrafish were induced. However, EUMF restored high expressions of *ambra1a*, *atg5*, *ulk1b*, and *lc3b* induced by AlCl_3_. Thus, autophagy was not excessively activated. Accordingly, this reduced the extracellular Aβ plaque count and reversed AD’s disease-like pathology in zebrafish.

*Ache* is the gene that encode Ache that inactivates the neurotransmitter ACh by catalyzing its hydrolysis to choline and acetic acid (Hu et al., 2019). *Slc6a3* is the gene that encode the dopamine transporter (Dat) that can provide rapid clearance of dopamine (DA) (Dedic et al., 2018). The primary function of ACh is to complete the transmission of neural signals. Once the synthesis and decomposition of ACh is abnormal, neural signaling transition may be blocked. To some extent, AD will be the result (Hu et al., 2019). DA is also a neurotransmitter that is critically implicated in cognitive function. Previous studies have found that the restoration of DA transmission plays a role in learning and memory in the mouse model of AD. DA dysfunction has a pathogenic role in the cognitive decline symptoms of AD (Martorana and Koch, 2014). Because Ache and Dat are inhibitors of ACh and DA, respectively, it is conceivable that they also play a critical role in the occurrence of AD. Interestingly, our results showed that both the expressions of *ache* and *slc6a3* genes were upregulated in the AD zebrafish model. However, treatment with EUMF reduced these increased expressions. Collectively, we suggest that beside of inhibiting the abnormal excessive autophagy, EUMF also reverse AD-like pathology in zebrafish by regulating the expressions of *ache* and *slc6a3* at the transcript levels. Definitely, we will perform gene expression test of other neurotransmitters including glutamate in the future to further investigate the underlying mechanism.

Flavonoids are a group of plant metabolites which can improve the cognitive functions. They can work within the processes associated with AD (Kaur et al., 2022; Maccioni et al., 2022). For example, quercetin belonging to the subcategory of flavonoids can significantly mitigate memory deficits in scopolamine mice model via protection against neuroinflammation and neurodegeneration (Olayinka et al., 2022). Eriodictyol which is a natural flavonoid compound can ameliorate cognitive dysfunction in APP/PS1 mice by inhibiting ferroptosis (Li L. et al., 2022). Anthocyanins can reduce the neuronal damage in *in vivo* and *in vitro* models of AD (Li H. et al., 2022). Here, we identified many flavonoids including quercetin-3′-O-glucoside, isoquercitrin, rutin hydrate, rutin, spiraeoside, isotrifoliin, isorhamnetin-3′-O-neohespeidoside, naringenin, naringenin chalcone, butin, myricitrin, isomucronulatol-7-O-glucoside, hyperoside, hesperetin 5-O-glucoside, narcissoside, di-O-methylquercetin, lonicerin, and morin in EUMF. Therefore, flavonoids in EUMF may contribute to its anti-AD effects. However, one limitation of this study is that the exact compounds of flavonoids in EUMF, which act as a promising agent against AD need further investigation. In the further work, we will analyze the composition and activity of the flavonoid compounds in EUMF to thoroughly understand the anti-AD activity of EUMF.

## Conclusion

**FIGURE 7:**
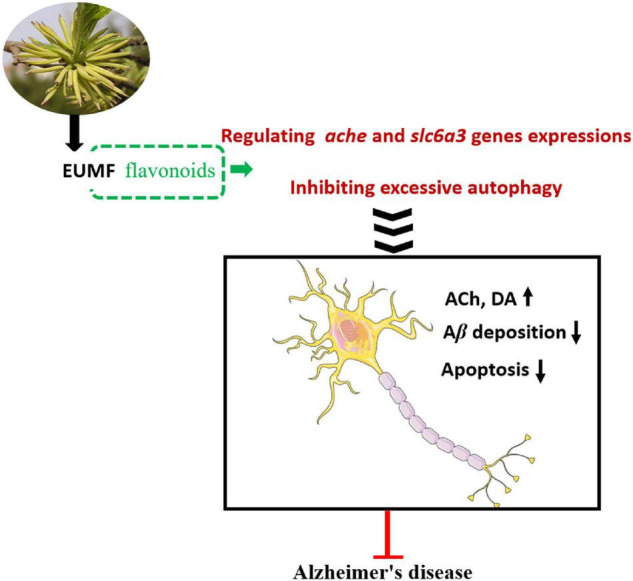
The proposed mechanism underlying the anti-AD effect of EUMF. EUMF inhibits the excessive autophagy and abnormal expressions of the *ache* and *slc6a3* genes to exert the therapeutic effects against AD-like symptoms. Flavonoids in EUMF may contribute to this biological process.

In conclusion, our study provided evidence that EUMF had anti-AD activity. EUMF ameliorated AD-like pathology in zebrafish possibly by inhibiting excessive autophagy and the abnormal expressions of *ache* and *slc6a3.* Flavonoid compounds in the EUMF may contribute to this biological process (Figure 7). Our data implied that EUMF is an attractive therapeutic candidate for AD.

## Data Availability Statement

The original contributions presented in the study are included in the article/supplementary material, further inquiries can be directed to the corresponding author.

## Ethics Statement

The animal study was reviewed and approved by the Animal Ethics Committe of Biology Institute, Shandong Academy of Sciences.

## Author Contributions

MJ conceptualized the idea and supervised the entire study. CS, SZ, SB, and JD performed the study and analyzed the results. MJ, QR, and YZ analyzed the results. CS and SZ wrote the manuscript. MJ and KL revised the manuscript and contributed to the final form. All authors read and approved the final manuscript.

## Conflict of Interest

The authors declare that the research was conducted in the absence of any commercial or financial relationships that could be construed as a potential conflict of interest.

## Publisher’s Note

All claims expressed in this article are solely those of the authors and do not necessarily represent those of their affiliated organizations, or those of the publisher, the editors and the reviewers. Any product that may be evaluated in this article, or claim that may be made by its manufacturer, is not guaranteed or endorsed by the publisher.
